# Persistent IgG1 clones dominate and personalize the plasma antibody repertoire

**DOI:** 10.1126/sciadv.adt7746

**Published:** 2025-04-16

**Authors:** Danique M. H. van Rijswijck, Albert Bondt, Dina Raafat, Silva Holtfreter, Kilian A. Wietschel, Sjors P. A. van der Lans, Uwe Völker, Barbara M. Bröker, Albert J. R. Heck

**Affiliations:** ^1^ Biomolecular Mass Spectrometry and Proteomics, Bijvoet Center for Biomolecular Research and Utrecht Institute for Pharmaceutical Sciences, University of Utrecht, Padualaan 8, Utrecht 3584 CH, Netherlands.; ^2^ Netherlands Proteomics Center, Padualaan 8, Utrecht 3584 CH, Netherlands.; ^3^ Institute of Immunology, University Medicine Greifswald, Greifswald, Germany.; ^4^ Department of Microbiology and Immunology, Faculty of Pharmacy, Alexandria University, Alexandria, Egypt.; ^5^ Department of Medical Microbiology, University Medical Center Utrecht, Utrecht University, Utrecht, Netherlands.; ^6^ Department of Functional Genomics, Interfaculty Institute for Genetics and Functional Genomics, University Medicine Greifswald, Greifswald, 17475, Germany.

## Abstract

Antibodies play a pivotal role in the immune defense and long-term immunity. Yet, while several studies have highlighted the persistence of antigen-specific antibody responses, it is unclear whether this stems from the continuous production of the same clones or recurrent activation of B cells generating new clones. To examine the stability of the human antibody repertoire, we monitored the concentrations of the most abundant IgG1 clones in plasma samples of 11 healthy donors at nine sampling points over a year. During this year, each donor received three doses of a COVID-19 vaccine. Notwithstanding these vaccinations, the concentrations of the most abundant IgG1 clones remained constant. Given the 2- to 3-week half-life of IgG1 molecules in blood, our data suggest that these clones are associated with long-term immunity and do not undergo somatic hypermutation which would imply short-lived plasma cells. Overall, our data suggest that most of the abundant IgG1 clones in plasma are persistently produced by long-lived plasma cells.

## Teaser

Abundant IgG1 clones in blood persist for at least a year, likely originating from long-lived plasma cells.

## INTRODUCTION

Antibodies play a key role in humoral immunity, an essential aspect of the adaptive immune response. Antibodies not only identify and neutralize pathogens but also play a crucial role in immune memory, allowing for rapid and effective responses to subsequent exposures with the same pathogen. B cells, responsible for the production of antibodies, originate from hematopoietic stem cells in the bone marrow. During their development, B cells are initially characterized by the expression of a membrane-bound immunoglobulin, also known as the B cell receptor (BCR) (*1*–*3*). The BCR consists of a fragment crystallizable (Fc) region which is membrane anchored and two fragment antigen-binding (Fab) regions. The maturation of B cells first takes place in the bone marrow and is continued and finalized in secondary lymphoid organs (*3*–*5*). The Fab regions of each BCR are highly diverse in sequence, resulting from somatic recombination of variable (V), diversity (D), and joining (J) gene segments, which forms the basis of the combinatorial diversity of antibodies. This diversity is further enhanced by nucleotide sequence changes at the junctions where these gene segments recombine, known as junctional diversity (*6*). The parts of the Fab where most diversity occurs are termed as the complementarity-determining regions (CDRs) because they together form the antigen-binding site, which is complementary to the antigenic epitope. This diversity in CDR sequences guarantees that almost any antigen can be targeted by particular B cells in the naïve repertoire (*3*, *7*). When B cells bind to an antigen they are activated, the clones expand, and the antibody-encoding genes can undergo additional rounds of modification through class-switch recombination and somatic hypermutation, to further fine-tune the Fc and Fab regions, respectively (*8*–*10*, *3*, *6*, *11*). B cells compete with one another for antigen binding, creating selection pressure that favors survival and further diversification of B cells with the highest affinity for the antigen. Over time, this process of directed molecular evolution increases the average affinity of the antigen-specific B cell and antibody repertoire, which is known as affinity maturation of the humoral immune response (*12*, *13*). Each combination of Fc and Fabs forms a unique antibody clone, which has its unique protein sequence and thus mass.

After a B cell is activated—and in some cases further modified to proficiently target the specific antigen—it starts to proliferate, and the daughter cells differentiate into either memory B cells or plasma cells (*14*, *15*). While memory B cells carry BCRs that are highly tuned to recognize and respond to repeated encounters with the same antigen, plasma cells are effector cells that continuously produce and secrete their BCRs in the form of antibodies into circulation. With all these possible diversifications in genomic rearrangements, somatic hypermutations, and posttranscriptional processing, the number of conceivable unique antibody clones is tremendous, with estimates reaching up to a quintillion (10^18^) (*16*), although the exact number our body may be able to generate is still an open and highly debated question. Through our recent work, we observed that the clonal repertoire as present in our blood as antibodies is possibly much more restricted and dominated by several hundreds of antibody clones, meaning that only a few clone account quantitively for the majority of the detected repertoire (*17*, *18*). Following up on these initial findings, here, we further characterize personalized plasma antibody repertoires, now over a substantially extended period of about 1 year, to improve our understanding of their origin, persistence over time, and function in the humoral immune response.

Previous studies on the persistence of antibodies over time primarily focused on either analyzing memory B cells (*19*) or tracking total antigen-specific antibody levels in blood over time, often using enzyme-linked immunosorbent assays (ELISAs) (*14*, *20*, *21*). In these studies, it was reported that antibody titers against specific viral antigens (e.g., measles, mumps, and rubella) remain stable in a donor’s blood over extensive periods, sometimes even throughout life (*14*, *20*, *21*). The half-life of most immunoglobulin G1 (IgG1) molecules in circulation is estimated to be, on average, 3 weeks, necessitating replenishment to sustain this observed long-term antibody-mediated immunity (*22*). Replenishment of antibodies can be facilitated by either memory B cells, which rapidly differentiate into antibody-producing plasma cells upon reexposure to their antigens, or by long-lived plasma cells (LLPCs), which continuously secrete antibodies for long-term immunity. Unlike their short-lived plasma cell (SLPC) counterparts, which have a brief life span and produce antibodies for immediate response, LLPCs can persist for years and primarily reside in the bone marrow (*15*, *23*, *24*).

Previous studies on antibody persistence over time lack details on the persistence of individual clones, which is essential to determine the origin and longevity of specific B cell clones. The liquid chromatography–mass spectrometry (LC-MS)–based Fab profiling approach used here allows us to resolve antibody heterogeneity and simultaneously measure the concentration of hundreds of individual antibody clones in blood. In this approach, we use a protease that specifically cleaves all IgG1 molecules captured from blood just above their hinge region. This allows us to examine and mass analyze the variable Fab regions of each clone that contain all the CDRs responsible for the diversity of antibody specificity and of antigen binding (*17*). Using this approach, we aimed to investigate the persistence of antibody repertoires in the plasma of 11 healthy donors, sampled nine times over a period of 1 year. During this year, each donor received three doses of a COVID-19 vaccine (Pfizer-BioNTech, BNT162b2). Notwithstanding the strong stimulus posed by these vaccinations, we observe that the clones dominating the IgG1 repertoire in the blood of healthy donors remain extremely constant over time in both concentration and sequence. This suggests that these most dominant class-switched IgG-producing clones do not undergo further somatic hypermutation or affinity maturation during this entire year. Mutations would be expected if they were the results of an ongoing germinal center reaction or the reactivation of memory B cells (*25*). The persistent occurrence of these most abundant plasma IgG antibodies differs deeply from those produced as a consequence of vaccination against the spike protein of severe acute respiratory syndrome coronavirus 2 (SARS-CoV-2) (*26*). All our participants were immunologically naïve to SARS-CoV-2 at the beginning of the study, and their antibody response to the vaccination (three doses) was characterized by rapid titer changes (up and down) over several logs. In addition, the antibody fine specificity changed, hinting at ongoing somatic hypermutation (*26*). Similar observations have been reported from other studies (*27*, *28*). Therefore, the most likely explanation for the clonal persistency we observe here is that these most abundant antibody clones are consistently produced at the same levels by LLPCs (*25*). Furthermore, the responsible LLPCs seem to neither be displaced from the finite LLPC niche by newly induced emerging clones of different specificity nor do the newly induced PCs become extensively predominant (*23*).

## RESULTS

### Study cohort used for longitudinal plasma IgG1 Fab profiling

**Fig. 1.**
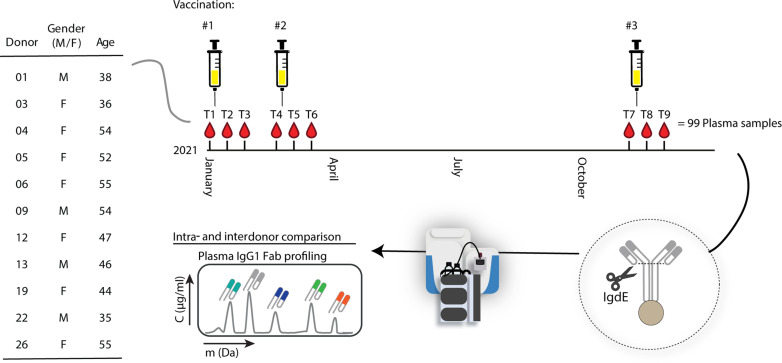
Study overview. Blood samples were collected from 11 donors at nine different time points (T1 to T9) over the course of a year (2021). Each of the donors received three doses (#1 to #3) of the COVID-19 vaccine. From a total of 99 plasma samples (11 donors × 9 time points), 10 μl of plasma was extracted, from which the IgGs were affinity captured. The protease IgdE was applied to digest and elute the Fab fragments exclusively from IgG1. The resulting mixture of Fab fragments was subsequently subjected to LC-MS at the intact Fab level. Each LC-MS peak, exhibiting a specific mass and RT pair, was considered a unique clone and annotated as ^RT^#_mass_. By spiking in recombinant mAbs at known concentrations, the concentrations of all detected individual plasma clones could be determined. M, male; F, female.

To investigate the persistence of individual IgG1 clones in blood, we analyzed the IgG1 clonal repertoires of 11 healthy donors (seven females/four males between 35 and 55 years of age) over the course of 1 year (Fig. 1). Sampling was carried out as part of a longitudinal COVID-19 vaccination study in 2021 during the Corona pandemic. All included participants received three doses of the COVID-19 mRNA vaccine BNT162b2 (Comirnaty, Pfizer-BioNTech). Notably, the participants were SARS-CoV-2 naïve at the time of enrollment (*26*). For each healthy donor, blood was collected at nine different time points: one sample just before each of the three vaccinations, one sample 7 days after each vaccination, and one sample 14 days after each vaccination (Fig. 1). The first two vaccinations were administered within 1 month, resulting in the first six sample points being collected within the first 2 months. The third booster vaccination was given approximately 9 months after the second dose, creating a time interval of about a year between the sampling of the first and the final sample. In total, this study included 99 blood samples (11 donors × 9 time points), from which we retrieved the plasma fractions. From these plasma samples, IgGs were affinity-purified using a high-affinity resin. Subsequently, the IgG1-specific protease IgdE was used to release the Fab fragments, which were then qualitatively and quantitatively profiled using LC-MS (Fig. 1). This cohort of longitudinally sampled healthy donors enabled us to analyze and monitor the individual IgG1 Fab repertoires over time with clonal resolution, allowing us to investigate the changes over time and assess the potential impact of the COVID-19 vaccination. We compared the IgG1 Fab repertoires both intra- and inter-individually. Here, each detected IgG1 antibody clone is classified by its unique combination of LC retention time (RT) and accurate mass (Da) and annotated with the clone ID ^RT^#_mass_. This enables the identification of modifications to a clone resulting from somatic hypermutation, through consequential changes in mass and/or RT. In addition, by spiking in recombinant IgG1 monoclonal antibodies (mAbs) at known concentrations, the concentration of each endogenous clone can be determined. Previously, we demonstrated the repeatability of this LC-MS–based clonal profiling method, both in terms of technical and sample preparation replicates (*17*).

### Each donor’s plasma IgG1 repertoire provides a unique longitudinal fingerprint

**Fig. 2.**
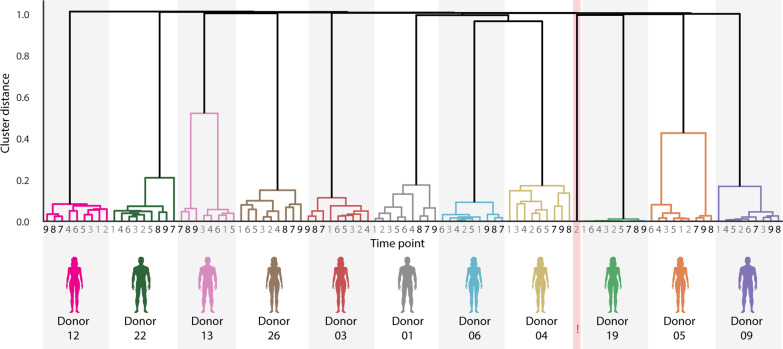
Quantitative longitudinal plasma IgG1 clonal repertoires provide donor-specific “fingerprints.” Concentration- and mass-based hierarchical clustering of the 99 Fab clonal repertoires displayed in fig. S1 (A to K). The nine timepoints on the *x* axis for each donor correlate to the nine sampling points as indicated in Fig. 1. In the cluster diagram, branch lengths represent the distances between repertoires. While no meaningful clustering was observed between different donors, repertoires from the same donor clustered tightly, even over the time span of a year, and notwithstanding the fact that each donor received three doses of COVID-19 vaccinations during this period. Notably, the second time point of donor 13 (annotated with a “!”) does not cluster with any other samples. Consequently, on the basis of its unique “fingerprint,” it was concluded to be a mislabeled sample, originating from an unknown donor, and thus excluded from further analysis.

As described previously, the LC-MS data acquired for each sample can be converted into quantitative mass clonal profiles, reflecting the IgG1 repertoires. These 99 IgG1 Fab clonal profiles are displayed in fig. S1 (A to K). In addition, we estimated the total detected IgG1 concentration, calculated by summing the individual concentrations of all detected clones per sample, as presented in table S2. While this may underestimate the actual IgG1 levels in the samples, it did reveal relatively consistent levels per donor. Next, hierarchical clustering, based on clone IDs and intensities, was used to cluster all 99 quantitative clonal repertoires (Fig. 2). This analysis revealed that the nine IgG1 repertoires from a single donor cluster closely together, while IgG1 repertoires from different donors do not cluster together at all. The IgG1 Fab clonal profiles shown in fig. S1 (A to K), along with the clustering results shown in Fig. 2, clearly reveal that the qualitative and quantitative IgG1 Fab repertoire within a specific donor remains relatively stable over a period of around 1 year. This reveals that most of the abundant IgG1 clones present on the first day of sampling are still being produced almost a year later and are present in plasma at comparable concentrations. In addition, all detected abundant IgG1 clones (i.e., ^RT^#_mass_) are unique to each individual, consistent with earlier reported data (*17*). Upon closer examination, we found that, for a few donors, the last three samples (T7 to T9) do cluster more closely with each other than with the first six samples (e.g., donors 05 and 13) (Fig. 2). In these two donors, there is thus a substantial change in the repertoire, but only after a relatively long interval of about 9 months without sampling, between the 6th and 7th time points.

Another notable observation is that the Fab repertoire obtained at the second time point of donor 13 (annotated with a “!” in Fig. 2) neither clusters with the other time points of donor 13 nor with any sample from the other donors. We conclude that this sample has been mislabeled, reinforcing the postulation that IgG1 Fab profiles measured over time can serve as a fingerprint for a certain donor. We consequently excluded the sample from the second time point of donor 13 from further analysis. Summarizing, the data shown in Fig. 2 and fig. S1 (A to K) suggest that the plasma concentrations of the dominant IgG1 clones remain highly stable over the course of a year, creating a fingerprint for each individual.

Moreover, in most donors, the IgG1 clonal repertoire is dominated by a dozen of high abundant clones. These IgG1 molecules cumulatively account for around a quarter of the detected total IgG1 concentration, making these dozen of high abundant clones especially important for shaping the antibody fingerprint for each individual. Exceptionally, in donor 19, a single IgG1 clone contributes >70% to the detected total IgG1 concentration (figs. S1I and S2). Beyond these dozen dominating clones, the IgG1 Fab repertoire seems to become more polydisperse, see also fig. S2. The (lack of) polydispersity of the IgG1 repertoire in a given donor does not seem to change substantially over a year and thus also seems to be remarkably unaffected by the repeated COVID-19 vaccinations.

### The concentration of most abundant IgG1 clones remains constant over a period of at least a year, with a few notable exceptions

**Fig. 3.**
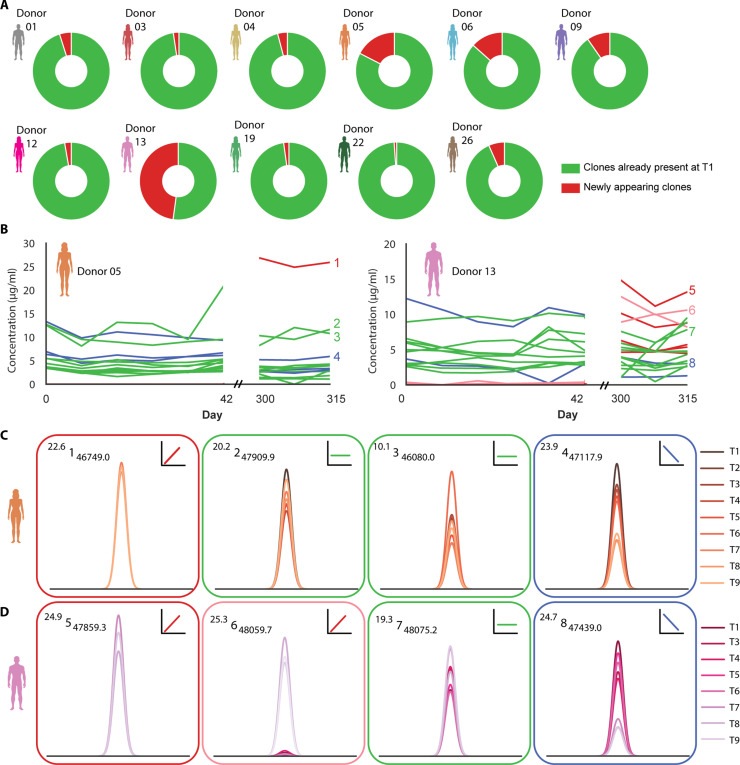
Stability and changes of IgG1 clonal abundances over a period of one year. (**A**) Pie charts for each donor illustrating the composition of the top 100 clones. Specifically, the composition of the total IgG1 concentration at T9 is shown, with the cumulative contribution of the newly appeared clones (i.e., clones not present at T1 to T3) indicated in red and the cumulative contribution of the clones already present at T1 indicated in green. For most donors, “newly” appearing clones contribute marginally to the repertoire. On the basis of these data donors 05 and 13 were selected for (B) to (D) as these displayed more substantial changes in the detected repertoire over time. (**B**) Variation in concentration over time of the 12 most abundant IgG1 clones at T1 and the top 12 most abundant clones at T9, with each line representing a unique clone. Clones were categorized on the basis of their trend in concentration: (i) red: newly appearing abundant clones at T7 and not detected at the six earliest time points; (ii) pink: clones that were initially present at low concentrations and became >30 times more abundant between T6 and T7; (iii) green: clones where the concentration remained stable over time; (iv) blue: clones abundant at the first six sampling points but more than twofold decrease in concentration between T7 and T9. (**C** and **D**) Magnified mass plots for each of the highlighted clones for donor 05 and donor 13, respectively. The peak color reflects the respective time point, and the border and sign of the mass plot illustrate the longitudinal behavior as categorized above. In the left-top corner of each panel, the unique clonal marker ^RT^ #_mass_ is depicted.

Focusing on the top dozen most abundant clones in each donor, we observed that these clones are present at all time points and that their individual concentrations remain relatively constant over the course of a year (fig. S1, A to K). This observed constancy in concentrations hints toward a very well balanced and long persisting IgG1 immunoglobulome. When examining the clonal repertoire, beyond these dozen most abundant clones, a small set of new clones does appear over time in some of the donors, albeit this is mainly observed after the long time interval (~9 months) between the sampling points of T6 and T7. These newly appearing clones contribute only marginally to the total IgG1 repertoire, with some notable exceptions, primarily for donor 05 and donor 13 (Fig. 3A). We identified a median of 5 newly appearing clones in the top 100 of T9 that were not observed in one of the first time points (T1 to T3), with one notable exception for donor 13 where we identified 46 newly appearing clones (Fig. 3A). Most of these newly appearing clones emerged after the ~9 months sampling interval (at T7) whereafter they remained stable in concentration over the last three time points (Fig. 3, B and D, red clones, and fig. S1H). In addition, a few clones that were already detected at very low concentrations at the first six sampling points increased more than 30 times in concentration at the last three sampling points (Fig. 3, B and D pink clones). In donor 05, one very abundant “new” clone emerged after the ~9-month sampling interval (at T7), which then remained abundant and stable in concentration over the last three sampling points (Fig. 3, B and C, red clone, and fig. S1D). However, also, in these donors, most of the initially dominant clones, as sampled at T1, remained remarkably constant in concentration over the period of one year (Fig. 3, green clones).

This study also allowed us to investigate the possible impact of the COVID-19 vaccination on the robustness and stability of the dominant IgG1 repertoire. Although a robust temporal antibody response against SARS-CoV-2 was measured in all donors after each vaccination (monitored by using a targeted Luminex assay; see fig. S3), indicating the generation of new vaccine-induced antibodies, this did not lead to substantial changes in the clonal profiles we measured. The COVID-19 vaccination stimulates the production of antigen-specific IgG clones, but their concentrations remain, in healthy donors, substantially lower than those of the dominant clones monitored within the IgG1 repertoire.

## DISCUSSION

As part of our humoral immune system, B cells may become activated by antigens, starting their differentiation into plasma cells that produce antibodies targeting specific antigens. These plasma cells secrete large quantities of antibody molecules that find their way into the circulation, defining the plasma antibody repertoire. Theoretically, every B cell can produce a unique antibody, and with an estimated 10^11^ B cells in our body, the antibody diversity in each person may be nearly infinite. However, here, and in agreement with our earlier studies (*17*), we observe that the plasma IgG1 antibody repertoire is, at the molecular level, dominated by just a few dozen to hundreds of clones. In this study, we build further upon these initial findings and reveal that the majority of the IgG1 clones retain their amino acid sequence and plasma concentration over a period of at least a year, although all donors in our cohort received three doses of a COVID-19 vaccine during this 1-year period.

Earlier studies focusing on the duration of humoral immunity have revealed that antibody titers against certain viral or bacterial antigens remain detectable in the blood of affected donors for extensive time periods, even up to a lifetime, for instance, following infection by measles, mumps, and rubella (*14*, *20*, *21*). It was suggested that the duration of antibody responses may depend not only on the antigen but also on now unknown host-specific factors. In the case of SARS-CoV-2, the titer tends to wane more quickly, with reported half-lives of 25 to 200 days (*29*–*36*). These studies typically determine the total antigen-directed IgG level in blood using ELISA-based assays. In contrast, in our study, we examine the persistence of individual IgG1 clones within the entire plasma antibody repertoire, enhancing our molecular understanding of the longevity of the most abundant IgG1 antibodies present in our blood. On the basis of the half-life of circulating antibody molecules in the blood, which is around 2 to 3 weeks for human IgG1, it can be inferred that, during a period of a year, all IgG1 proteins must have been turned over (*22*). Thus, the observed constancy in concentrations of individual IgG1 clones must stem from the continuous production of new antibody molecules of the same amino acid sequence. We propose that such a long-term stable response can be attributed either to constantly reactivated memory B cells or to the continuous production of specific IgGs by LLPCs (*23*). However, the involvement of memory B cells is less likely, as reactivation of these cells often leads to a cycle of somatic hypermutation resulting in amino acid sequence changes in the CDR loops, which underlies affinity maturation of the specific antibody repertoire (*13*). Each somatic hypermutation would manifest as changes in the masses of the Fabs detected by LC-MS and, in our approach, lead to their annotation as new clones. Therefore, long-term production of antibodies by LLPCs seems more plausible to explain our observations.

A key question is why the stability and dominance of B cell clones have not been previously detected like this, despite extensive studies on the B cell repertoire in large donor groups. The answer lies in the conventional approaches used to investigate antibody repertoires, which typically involve isolating peripheral blood B cells and sequencing their BCR genes from RNA or genomic DNA using multiplexed polymerase chain reaction. These approaches often focus on memory B cells (*37*, *38*) as these cells are most accessible. This focus leads to overlooking LLPCs, which primarily reside in the bone marrow and are difficult to access due to the invasive procedures required to retrieve them. However, we in our study overcome this limitation by analyzing directly the secreted antibodies present in blood, which likely originate from various types of plasma cells, including those that are typically less accessible like LLPCs.

It is assumed that, in each person, the LLPC niche is established early in life and that it becomes increasingly challenging for new B cells to access this niche when a person ages (*15*, *23*). Here, we observed that, in a few donors, a limited number of highly abundant clones emerged during the study period. The concentrations of these new clones appeared to be constant at the subsequent time points. Ideally, additional studies over even longer time periods are needed to find out whether these clones may originate from newly generated plasma cells that have conquered a place in the LLPC niche. Moreover, the reported challenge of newly generated plasma cells to enter or leave the LLPC niche when a person ages would imply that the clonal dominance could vary across different age groups, with elderly donors showing less dispersity in their clonal profile. The LLPC niche hypothesis would then also imply that the total plasma IgG1 antibody repertoire is primarily established early in life, remaining relatively constant thereafter. In our study, we observed a reasonable consistent polydispersity across all donors, ranging in age between 35 and 55 years. To investigate the formation of such a niche, we next should ideally profile IgG repertoires in newborns and infants over their first few months to years of life.

Another intriguing question is why our blood is dominated by such abundant and persistent antibody clones that are likely produced by LLPCs, and what they specifically target. Our study cannot address this question, but other studies suggested that the primary role of LLPCs is to provide sustained protective immunity against infrequent (but more severe) epidemic infections in an antigen-independent fashion, whereas SLPCs are primarily involved in protection against frequent (and less severe) endemic infections that is sustained by recurrent and antigen-dependent B cell reactivation (*39*). The specific targets of these antibodies (if any) require further investigation. In general, it is assumed that LLPCs produce high-affinity antibodies that are more affine than those generated by memory B cells (*15*, *24*). Moreover, whether a plasma cell makes it to an LLPC may be dependent on the involved bacterial or viral infection. It would therefore also be interesting to investigate whether infections such as measles, mumps, or rubella, which are known to lead to detectable antibodies in the blood over extensive periods, could be explained by some of these B cells differentiating into LLPCs.

Unexpectedly, our data revealed that COVID-19 vaccination did not substantially affect the concentrations of the most dominant clones in the IgG1 clonal repertoire. However, targeted assays clearly showed that all donors temporarily generated IgG antibodies against the SARS-CoV-2 spike protein, which they lacked before vaccination (*26*). Nonetheless, we did observe some changes among the less abundant clones in the repertoire. These findings align well with previous work, in which we examined both the complete plasma and SARS-CoV-2–specific IgG1 Fab repertoires in donors who had experienced a SARS-CoV-2 infection (*18*). In that study, we found that IgG1 antibodies specific to SARS-CoV-2 contributed only a small percentage of the total IgG1 concentration. Consequently, the SARS-CoV-2–specific clones were barely detectible within the total plasma IgG1 repertoire. This suggests that when examining the total plasma repertoires, we are only detecting the most abundant antibodies. Therefore, to accurately investigate antivaccine responses, examining only the total IgG1 repertoire is not sensitive enough, making a focus on antigen-specific IgG1 repertoires crucial (*18*, *40*). Our study thus clearly shows that clinically relevant protective antibodies, such as those elicited by repeated COVID-19 vaccination, may never enter the pool of dominant plasma antibody clones.

In conclusion, here, we report that the total IgG1 clonal repertoire in healthy donors remains quite constant over at least a period of a year, with barely any changes in the concentrations of the most dominant clones. Moreover, this longitudinally stable antibody repertoire signature is exquisitely unique for each donor. We thus conclude that these dominant clones do not undergo somatic hypermutation, making it most likely that they are produced by LLPCs. It remains to be seen whether this conclusion, that is based on solely monitoring IgG1 repertoires, is also valid for the other isotypes and subclasses of plasma antibodies. A more intriguing question that remains unanswered is what induces these abundant circulating antibodies and what antigen they target, which appear not to be induced by acute infections or vaccinations. It seems that we may maintain these same abundant antibody clones throughout our entire life span. The repertoire analysis approach used here therefore just offers first insights into the remarkable longevity of individual antibody clones within the antibody repertoire but raises also many more key immunological questions.

## MATERIALS AND METHODS

### Study cohort

AICOVI (Adaptive Immune Response to COVID-19 Vaccination) is a prospective clinical cohort study aiming at elucidating the kinetics of vaccine-specific antibody production after COVID-19 vaccination in health care workers at the Greifswald University Hospital, Germany (*26*). Participants were recruited before their intended vaccination. The selected subcohort received a homologous basic immunization with two doses of BNT162b2 [Comirnaty, tozinameran (INN), BioNTech/Pfizer] with a time interval of 4 weeks. Approximately 9 months later, participants received a third vaccination (homologous booster vaccination) with BNT162b2. Within the study, peripheral blood was collected by venipuncture on each day of vaccination as well as 7 and 14 days after each vaccination. From these blood drawings, EDTA plasma samples were prepared and stored at −20°C. The subcohort of 11 donors for the present study was carefully selected with respect to (i) complete vaccination schemes (3 × BNT162b2), (2) representative anti-S1 antibody profiles, (iii) sampling at the exact timepoints (day 0, day 7, and day 14 after vaccination), and (iv) no apparent deviations in terms of medication, chronic diseases, etc. All reported medications of the donors can be found in table S2, none of which should have had any effect on the antibody response/profiles to the vaccination or any infections.

The AICOVI study was approved by the Ethics Committee of the University Medicine Greifswald (internal registration numbers BB 001/21f) with all participants giving informed written consent. All work was conducted in accordance with the tenets of the Declaration of Helsinki (version 2013, Fortaleza). All requirements of data protection and confidentiality according to local regulations, the State Data Protection Act Mecklenburg-Western Pomerania, the European Data Protection Directive 95/46/EC, and the General Data Protection Regulation were fully met.

### Expression and purification of rIgdE

Cloning and protein expression were performed as previously described (*41*), with minor differences. In short, the amino acid sequence of IgdE was retrieved from UniProt (entry: A0A7Z7P3G8; gene name: NCTC8181_01479). The signal peptide sequence was removed, and using the online IDT Codon Optimalization Tool (Integrated DNA Technologies), the amino acid sequences were converted into a codon-optimized DNA sequence for expression in *Escherichia coli* K13. This optimized sequence was assembled as a gBlock (Integrated DNA Technologies) containing overhangs for insertion into a pRSET-C-His expression vector by Gibson Assembly (New England Biolabs). The assembled vector was transformed into *E. coli* BL21 (DE3) for protein expression, and rIgdE protein was purified under native conditions using affinity chromatography (5-ml HisTrap FF column, GE Healthcare). The bound protein was eluted on an Äkta Pure (GE Healthcare) using an imidazole gradient, dialyzed against phosphate-buffered saline, and stored at −80°C until further use.

### IgG capturing and subsequent IgG1 Fab generation

To capture IgG from plasma, a similar procedure was used as described previously (*17*, *42*). In short, 20 μl of CaptureSelect FcXL affinity matrix (Thermo Fisher Scientific) slurry was added to a Pierce Spin Column (Thermo Fisher Scientific) and washed three times with 150 mM phosphate buffer (PB). After washing, the affinity matrix was resuspended in 150 μl of PB containing two known IgG1 mAbs, trastuzumab (Roche, Penzberg, Germany) and alemtuzumab (Genmab, Utrecht, the Netherlands), at 200 ng each, and 10 μl of corresponding plasma. The samples were then incubated with the affinity matrix under shaking conditions for 1 hour at room temperature. After the incubation, the flow-through was collected, and the affinity matrix was washed four times with PB. To produce IgG1 Fab fragments, the affinity matrix was resuspended in 50 μl of PB containing 5 μg of IgG1-specific protease immunoglobulin degrading enzyme (IgdE, made in-house) and incubated for at least 16 hours at 37°C on a thermal shaker. The generated IgG1 Fab fragments were collected by centrifugation for 1 min at 500*g*. IgdE specifically cleaves human IgG1 just above the hinge region, yielding two Fab fragments per IgG1 molecule.

### LC-MS–based Fab profiling

To analyze the intact Fab fragments that were released, a reversed-phase LC-MS method and data processing were used, which were described previously (*17*). In short, Fab fragments were separated using a Vanquish Flex ultra high performance liquid chromatography (UHPLC) instrument (Thermo Fisher Scientific, San Jose, CA, USA) equipped with a 1–by–150 mm MAbPac Reversed Phase HPLC column (Thermo Fisher Scientific, CA, USA) and directly coupled to an Orbitrap Exploris 480 MS with BioPharma option (Thermo Fisher Scientific). Both the column preheater and the analytical column chamber were heated to 80°C, during chromatographic separation. Fab fragments were separated over a ~60-min gradient at a flow rate of 150 μl/min. Gradient elution used two mobile phases, A (0.1% HCOOH in MilliQ water) and B (0.1% HCOOH in CH_3_CN). The gradient started with a mixture of 90% A and 10% B, ramping up from 10 to 25% B over 1 min, from 25 to 40% B over 55 min, and from 40 to 95% B over the last minute of the gradient. MS data were collected in intact protein and low-pressure mode with the following settings: spray voltage: 3.5 kV; ion transfer tube temperature: 350°C; vaporizer temperature: 100°C; sheath gas flow: 15 arbitrary units; auxiliary gas flow: 5 arbitrary units; source induced dissociation: 15 V. Spectra were recorded with a resolution setting of 7500 [at 200 mass/charge ratio (*m/z*)] in MS1. Scans were acquired in the range of 500 to 4000 *m/z* using an automated gain control target of 300% and a maximum injection time of 50 ms. For each scan, 5 microscans were recorded.

### Fab profiling data analysis

To analyze the LC-MS results, the RT and mass (in dalton) of all intact Fab molecules were retrieved from the generated RAW files using BioPharmaFinder (BPF) 3.2 (Thermo Fisher Scientific), similar to the method described before (*17*). In short, deconvolution was performed using the ReSpect algorithm (Thermo Fisher Scientific) between 5 and 57 min using 0.1-min sliding windows with 25% offset and a merge tolerance of 30 parts per million (ppm). Noise rejection was set at 95% and the output range between 10,000 and 100,000 Da with a target mass of 48,000 Da and a mass tolerance of 30 ppm. Charge states between 10 and 60 were included, and the Intact Protein Peak model was selected.

Further data analysis was performed using in-house Python 3.9.13 scripts and the libraries Pandas 1.5.3 (*43*), Numpy 1.24.3 (*44*), Scipy 1.9.1 (*45*), Matplotlib 3.7.1 (*46*), and Seaborn 0.12.2 (*47*). Masses of the BPF identifications were recalculated using an intensity weighted mean, considering only the most intense peaks comprising 90% of the total intensity. Masses between 46.0 and 56.0 kDa with the most intense charge state above 1000 *m/z* and a BPF score of ≥40 were considered to be Fab fragments of IgG1 antibodies. Fab fragments were considered one clone when they fell within a predefined mass and RT windows, specifically 1.5 Da and 0.8 min, respectively. These windows were defined on the basis of the mAbs that were spiked in and some of the most abundant (stable) clones in each of the donors. Concentrations of detected Fab molecules were determined by normalizing their sum intensity to the averaged sum intensity of the internal mAb standard. The mAb standard was subtracted from the list of Fab fragments detected, and the remaining Fab fragments were defined as unique Fab molecules based on their unique pair of mass and RT.

### Luminex assay

Plasma IgG binding to SARS-CoV-2 Spike S1 was quantified with an in-house bead-based 10-plex suspension array based on the xMAP technology (Luminex, Austin, Texas, USA) as previously reported (*26*, *48*). The Luminex assay covered six recombinant SARS-CoV-2 antigens, including the S1 domain from the wild-type virus. The relative antibody concentration was estimated from the antibody titraton curves (plasma dilution, 1:20 to 1:312,500) using a nonlinear regression model (*49*).
