# Supplementary material for: Persistent IgG1 clones dominate and personalize the plasma antibody repertoire
Source: Sci Adv. 2025 Apr 16;11(16):eadt7746. doi: 10.1126/sciadv.adt7746 (PMC12002106; doi:10.1126/sciadv.adt7746)
Supplement: Supplementary file 1 — Figs. S1 to S3 Table S1 Legend for table S2 [file sciadv.adt7746_sm.pdf]

Science Advances
AAAS

Supplementary Materials for

Persistent IgG1 clones dominate and personalize the plasma antibody repertoire

Danique M. H. van Rijswijck *et al.*

Corresponding author: Albert J. R. Heck, a.j.r.heck@uu.nl

Sci. Adv. **11**, eadt7746 (2025)
DOI: 10.1126/sciadv.adt7746

The PDF file includes:

Figs. S1 to S3
Table S1
Legend for table S2

Other Supplementary Material for this manuscript includes the following:

Table S2

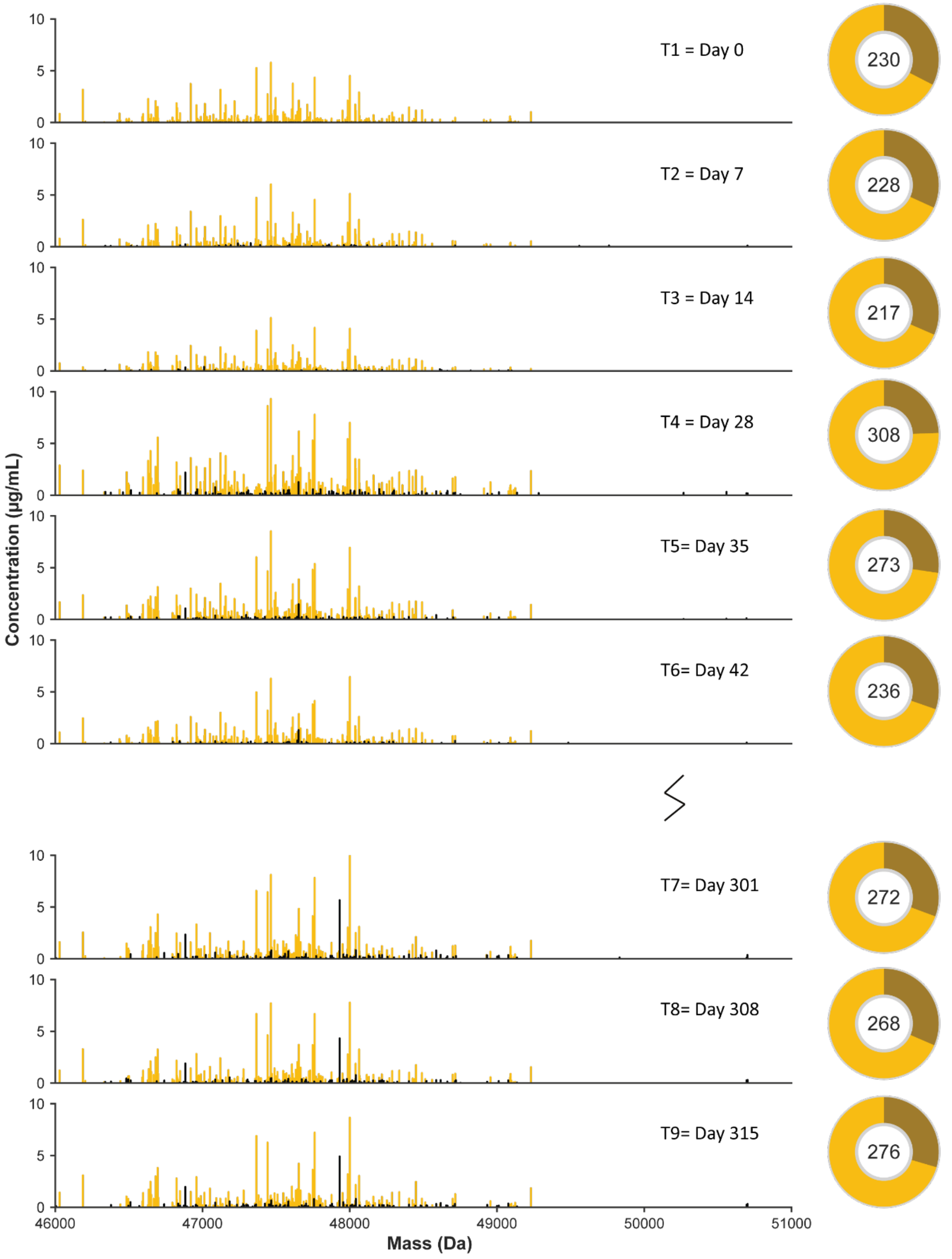

Fig. S1a. Longitudinal Fab clonal mass profiles of donor 01 at each of the 9 time points. Each peak represents a unique Fab at its detected mass and plasma concentration. The black clones are clones that were not present at Day 0. The pie charts display in the middle the total number of clones annotated, and the relative contribution of the top 12 (light brown) clones to the total IgG1 concentration (ocher yellow) in donor 01 at each time point.

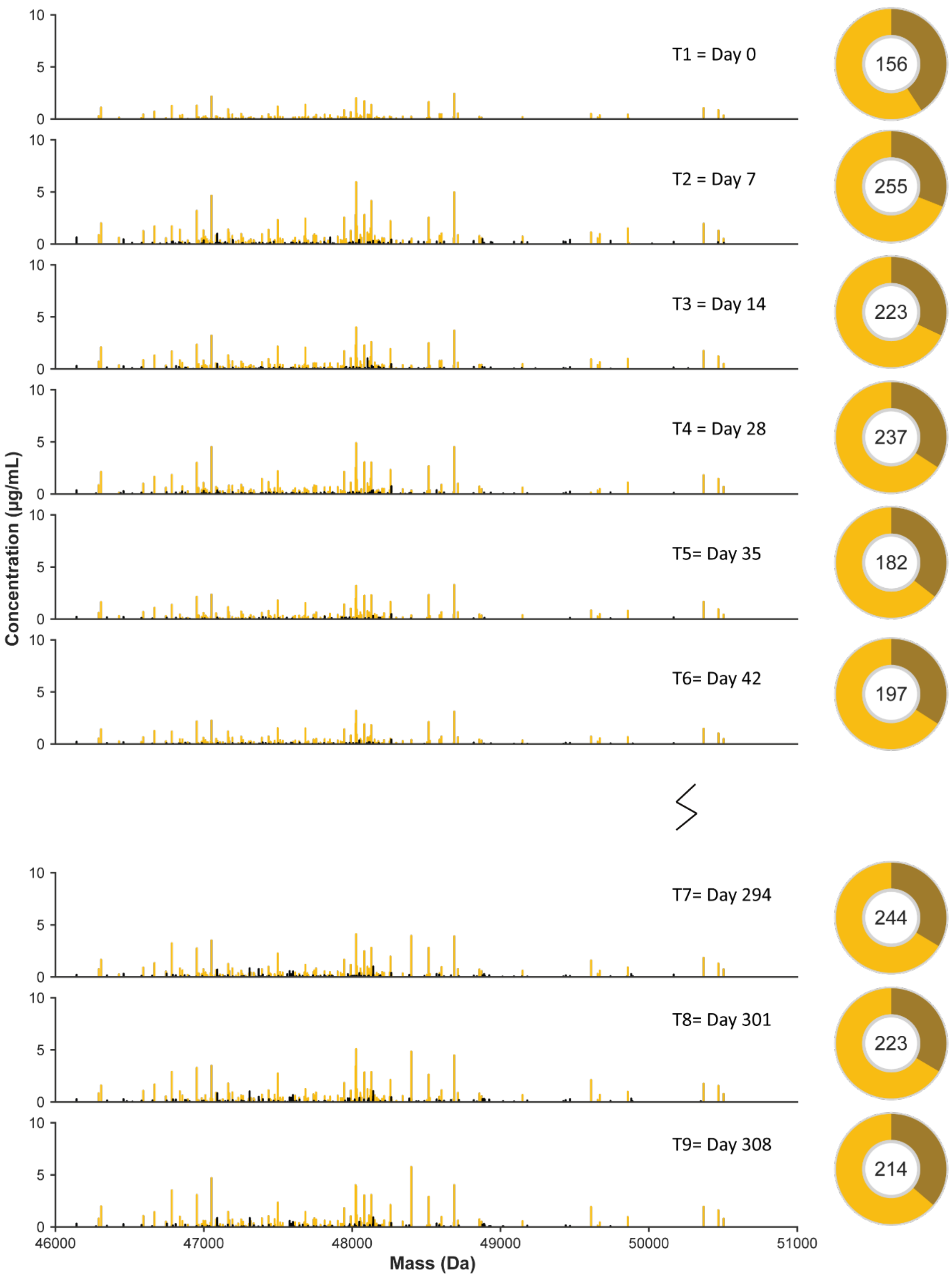

Fig. S1b. Longitudinal Fab clonal mass profiles of donor 03 at each of the 9 time points. Each peak represents a unique Fab at its detected mass and plasma concentration. The black clones are clones that were not present at Day 0. The pie charts display in the middle the total number of clones annotated, and the relative contribution of the top 12 (light brown) clones to the total IgG1 concentration (ocher yellow) in donor 03 at each time point.

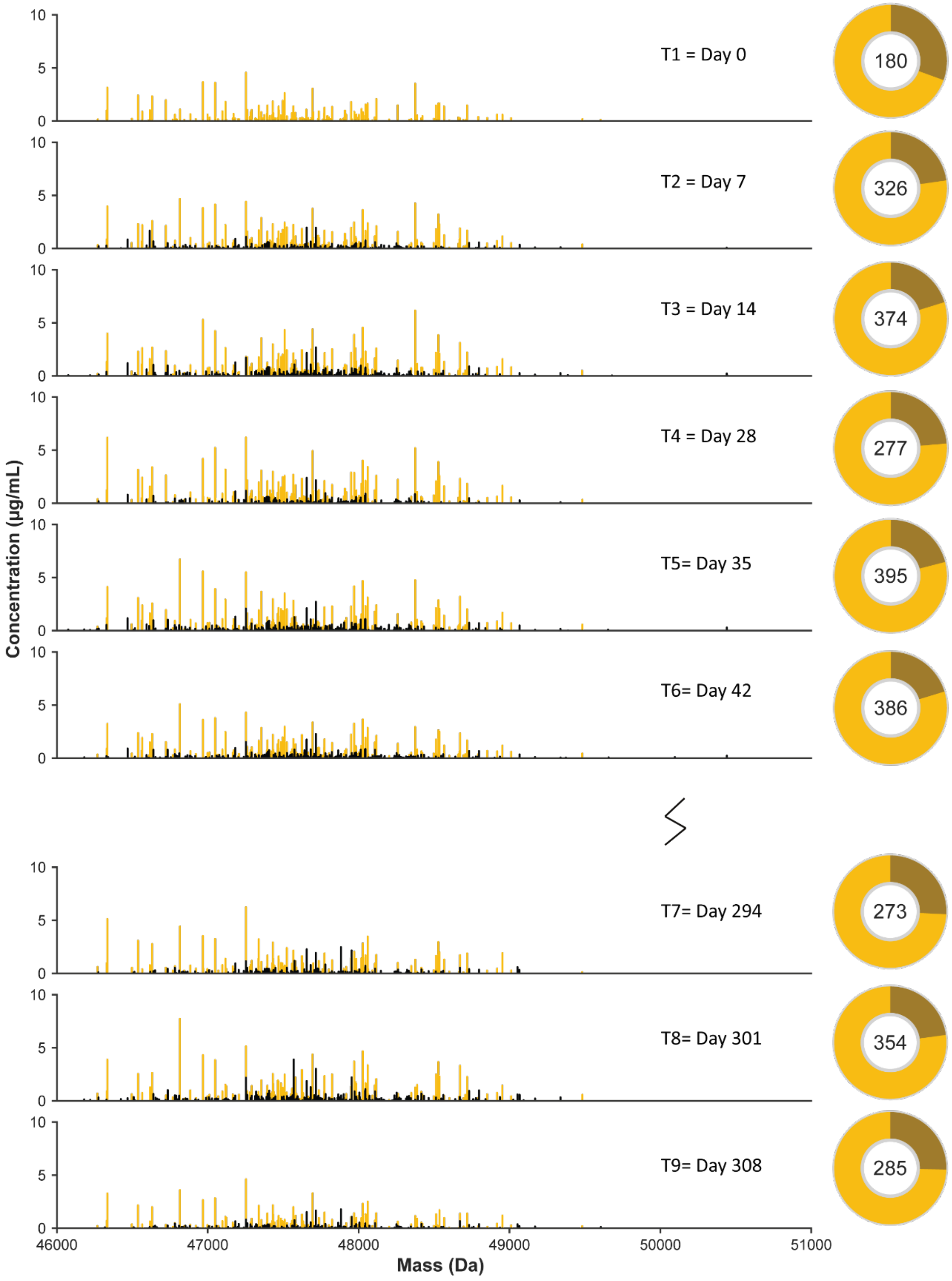

Fig. S1c. Longitudinal Fab clonal mass profiles of donor 04 at each of the 9 time points. Each peak represents a unique Fab at its detected mass and plasma concentration. The black clones are clones that were not present at Day 0. The pie charts display in the middle the total number of clones annotated, and the relative contribution of the top 12 (light brown) clones to the total IgG1 concentration (ocher yellow) in donor 04 at each time point.

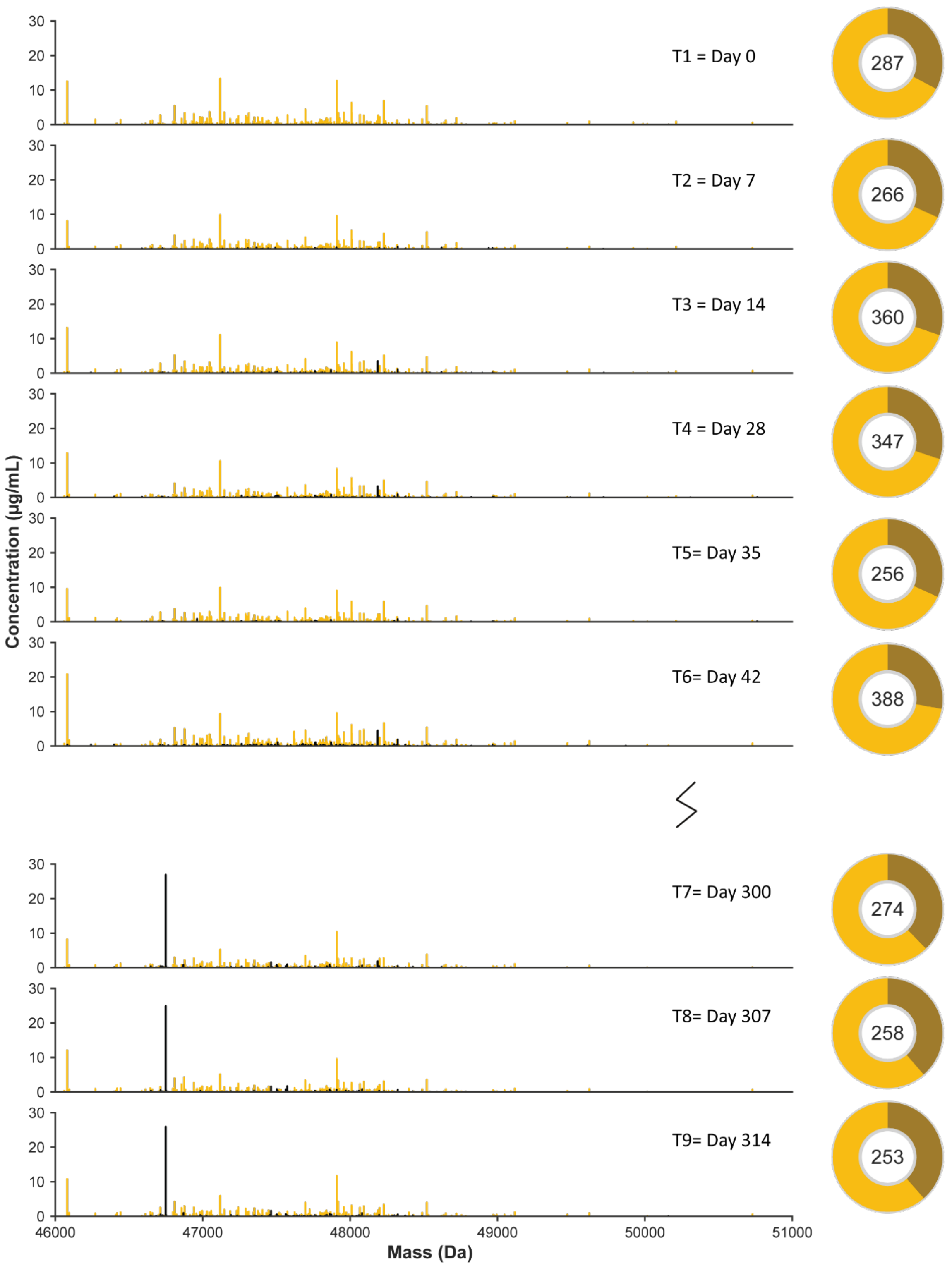

Fig. S1d. Longitudinal Fab clonal mass profiles of donor 05 at each of the 9 time points. Each peak represents a unique Fab at its detected mass and plasma concentration. The black clones are clones that were not present at Day 0. The pie charts display in the middle the total number of clones annotated, and the relative contribution of the top 12 (light brown) clones to the total IgG1 concentration (ocher yellow) in donor 05 at each time point.

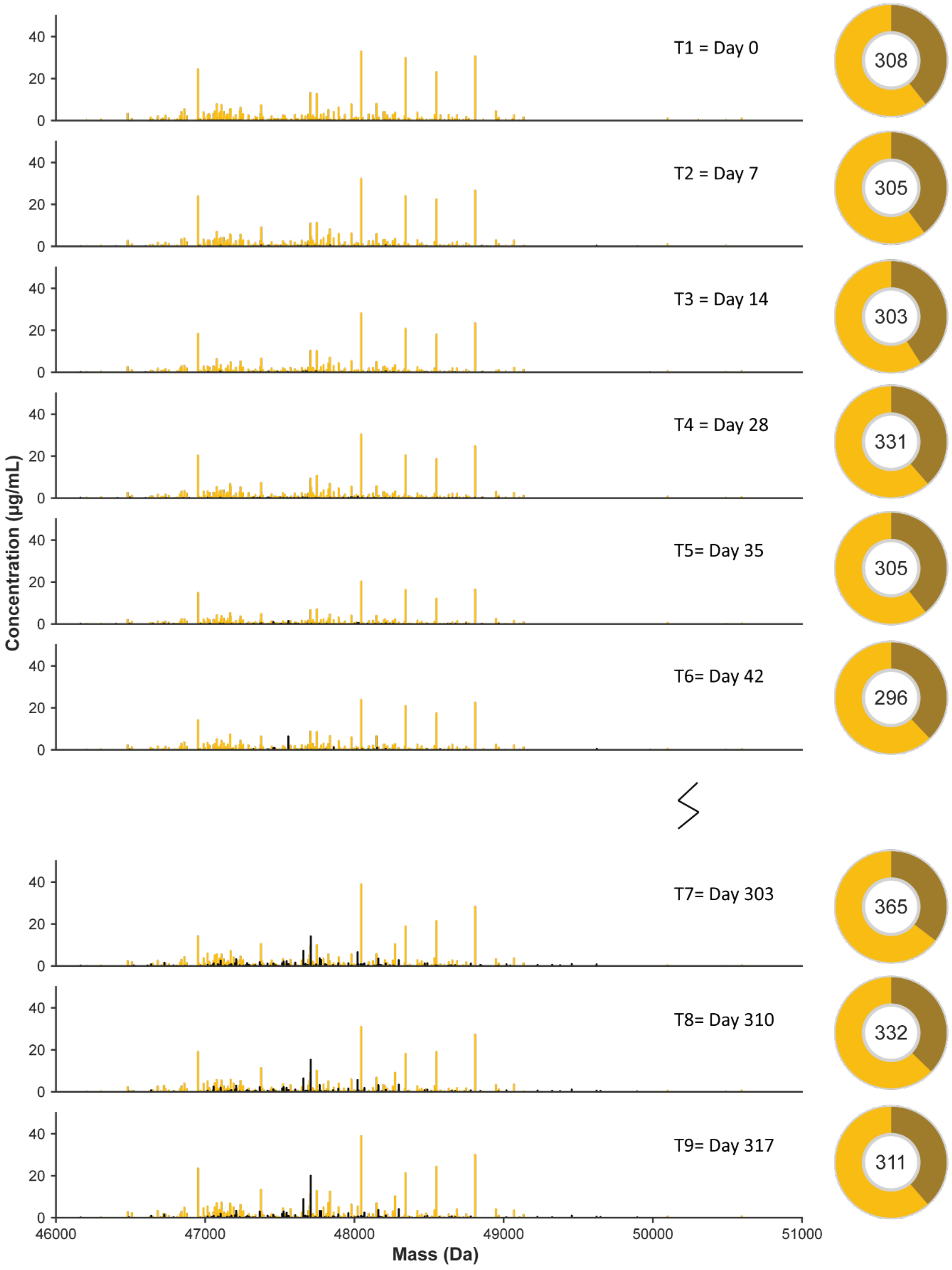

Fig. S1e. Longitudinal Fab clonal mass profiles of donor 06 at each of the 9 time points. Each peak represents a unique Fab at its detected mass and plasma concentration. The black clones are clones that were not present at Day 0. The pie charts display in the middle the total number of clones annotated, and the relative contribution of the top 12 (light brown) clones to the total IgG1 concentration (ocher yellow) in donor 06 at each time point.

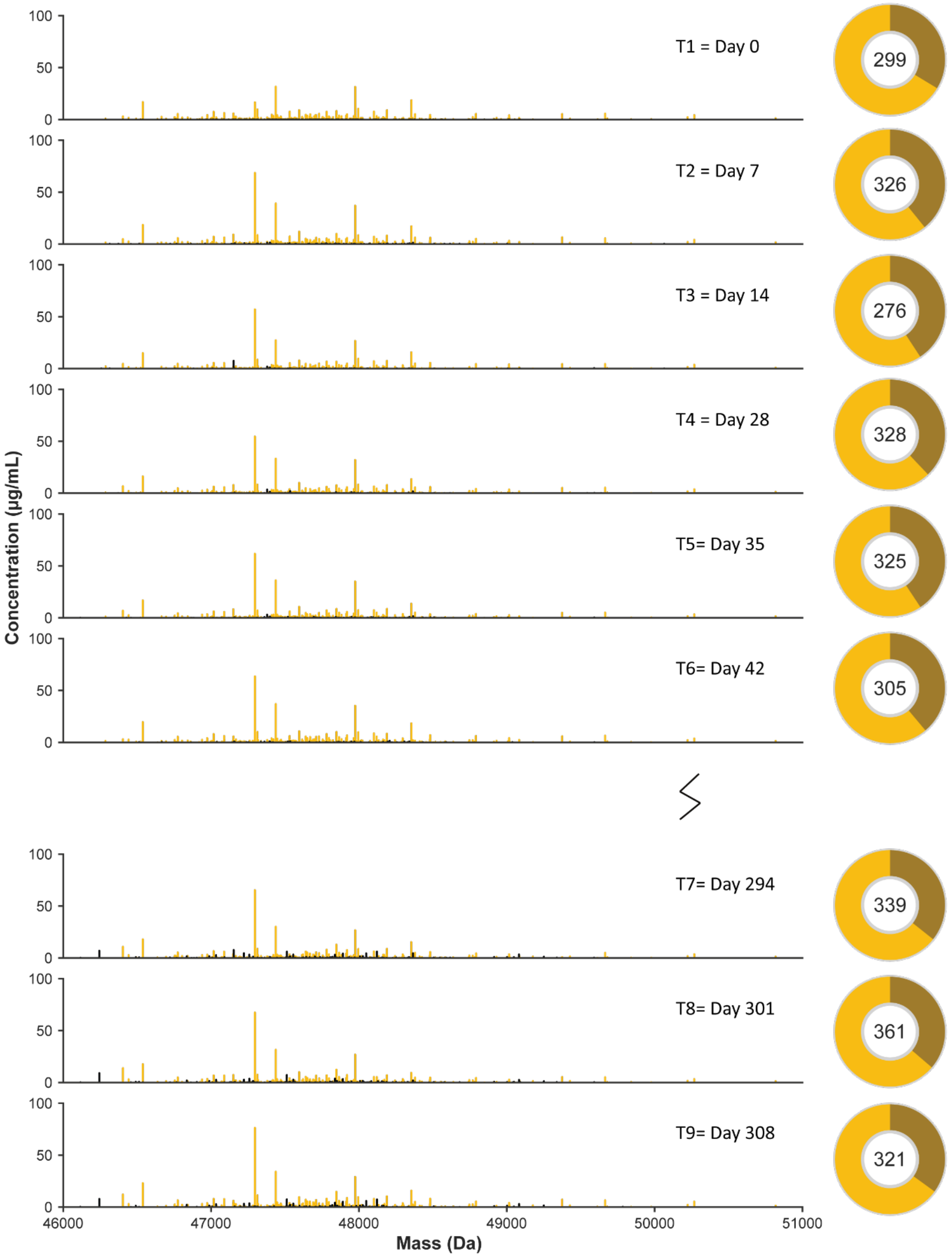

Fig. S1f. Longitudinal Fab clonal mass profiles of donor 09 at each of the 9 time points. Each peak represents a unique Fab at its detected mass and plasma concentration. The black clones are clones that were not present at Day 0. The pie charts display in the middle the total number of clones annotated, and the relative contribution of the top 12 (light brown) clones to the total IgG1 concentration (ocher yellow) in donor 09 at each time point.

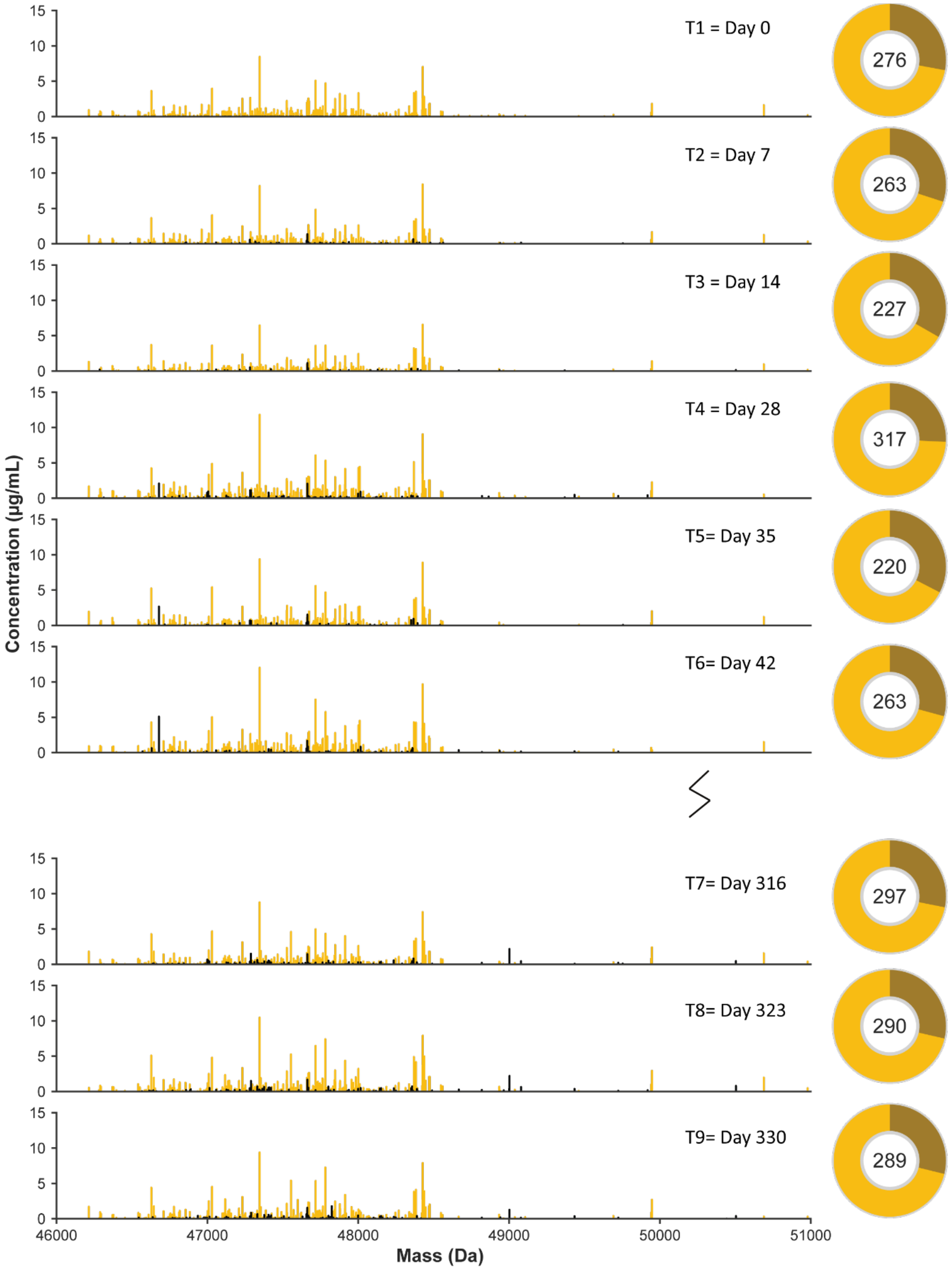

Fig. S1g. Longitudinal Fab clonal mass profiles of donor 12 at each of the 9 time points. Each peak represents a unique Fab at its detected mass and plasma concentration. The black clones are clones that were not present at Day 0. The pie charts display in the middle the total number of clones annotated, and the relative contribution of the top 12 (light brown) clones to the total IgG1 concentration (ocher yellow) in donor 12 at each time point.

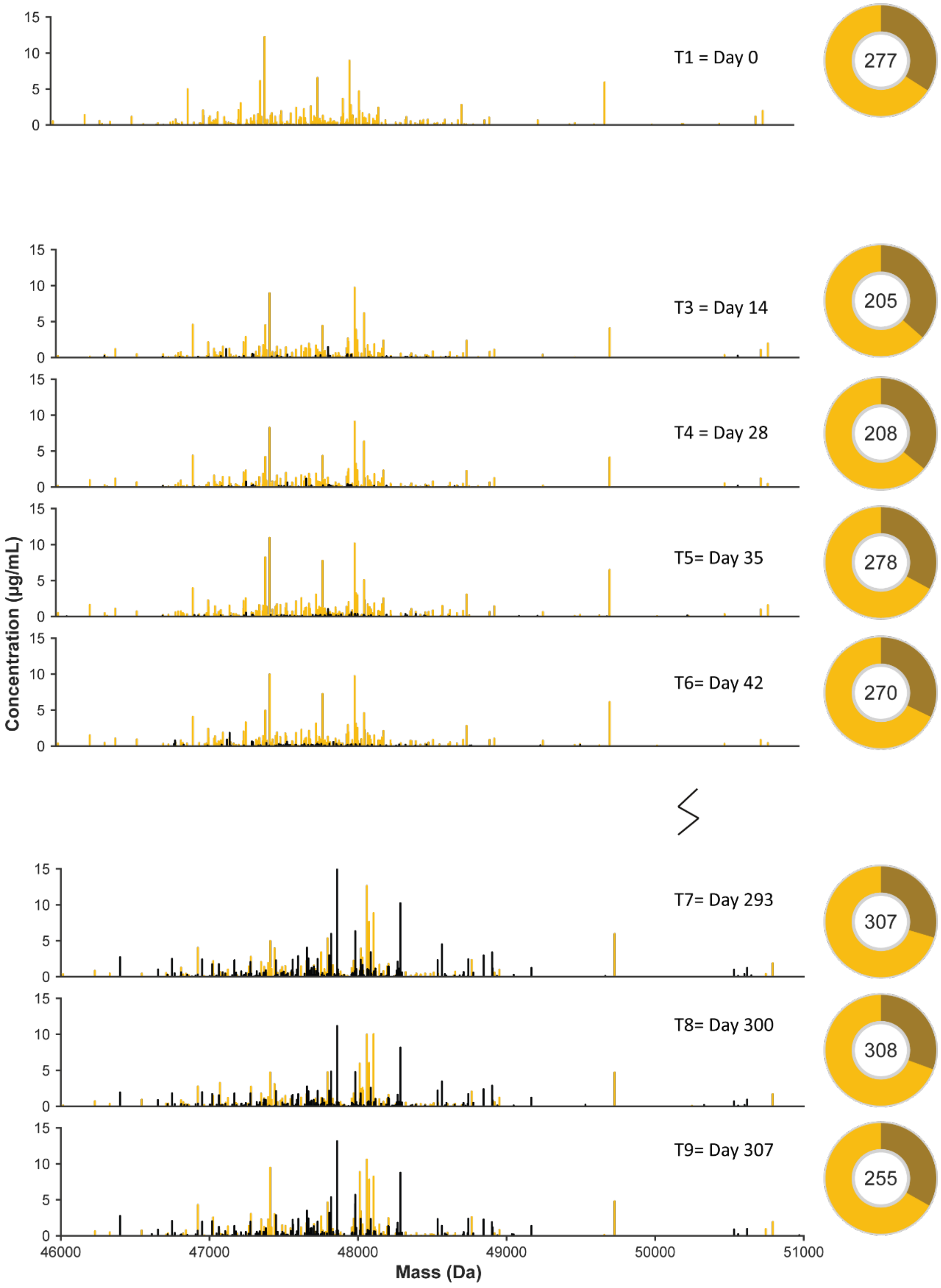

Fig. S1h. Longitudinal Fab clonal mass profiles of donor 13 at each of the 9 time points. Each peak represents a unique Fab at its detected mass and plasma concentration. The black clones are clones that were not present at Day 0. The pie charts display in the middle the total number of clones annotated, and the relative contribution of the top 12 (light brown) clones to the total IgG1 concentration (ocher yellow) in donor 13 at each time point. Of note: Time point 2 of this donor was a mislabeled sample and therefore excluded for further analysis.

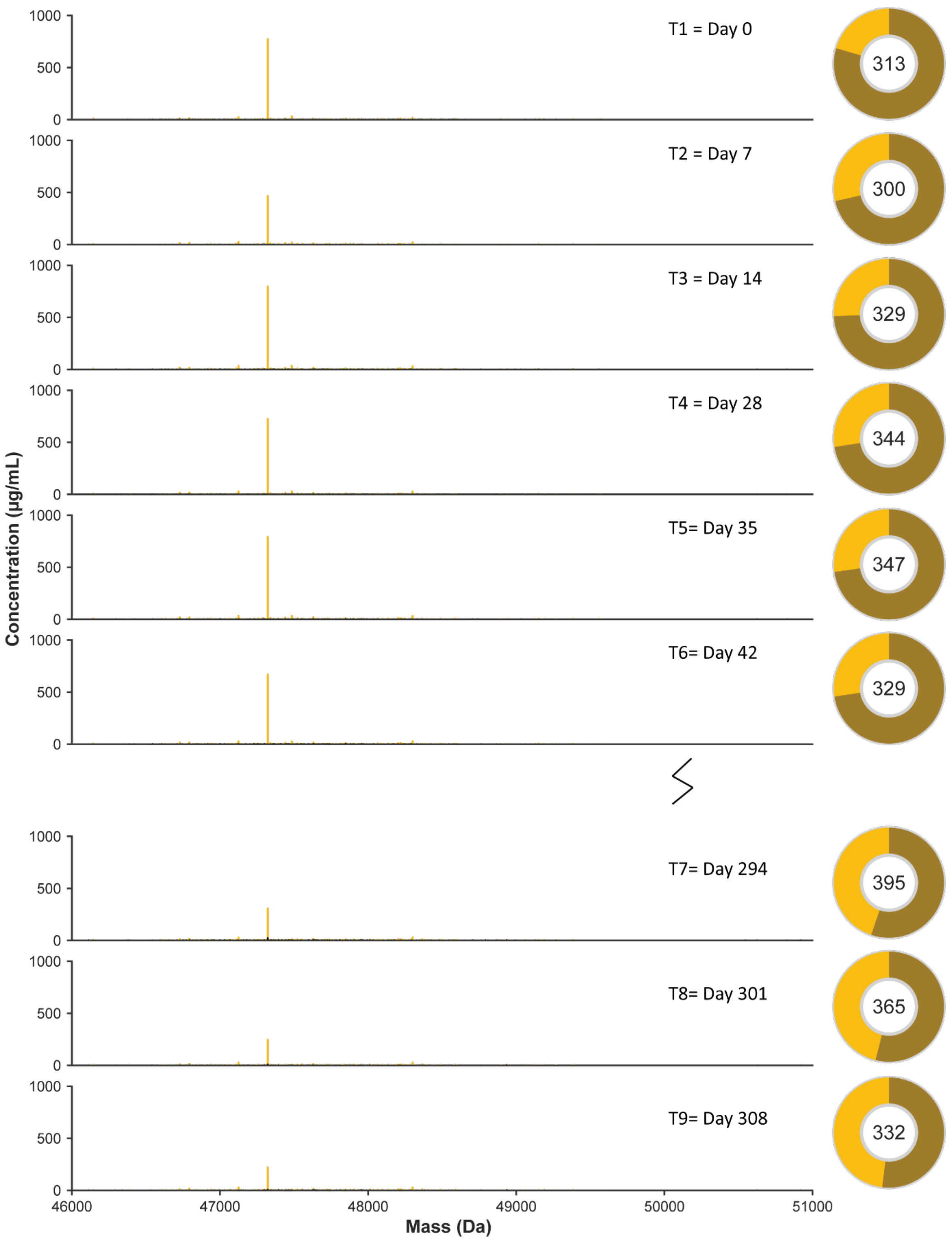

Fig. S1i-1. Longitudinal Fab clonal mass profiles of donor 19 at each of the 9 time points. Please note the exceptionally large range of the y-axis. Each peak represents a unique Fab at its detected mass and plasma concentration. The black clones are clones that were not present at Day 0. The pie charts display in the middle the total number of clones annotated, and the relative contribution of the top 12 (light brown) clones to the total IgG1 concentration (ocher yellow) in donor 19 at each time point.

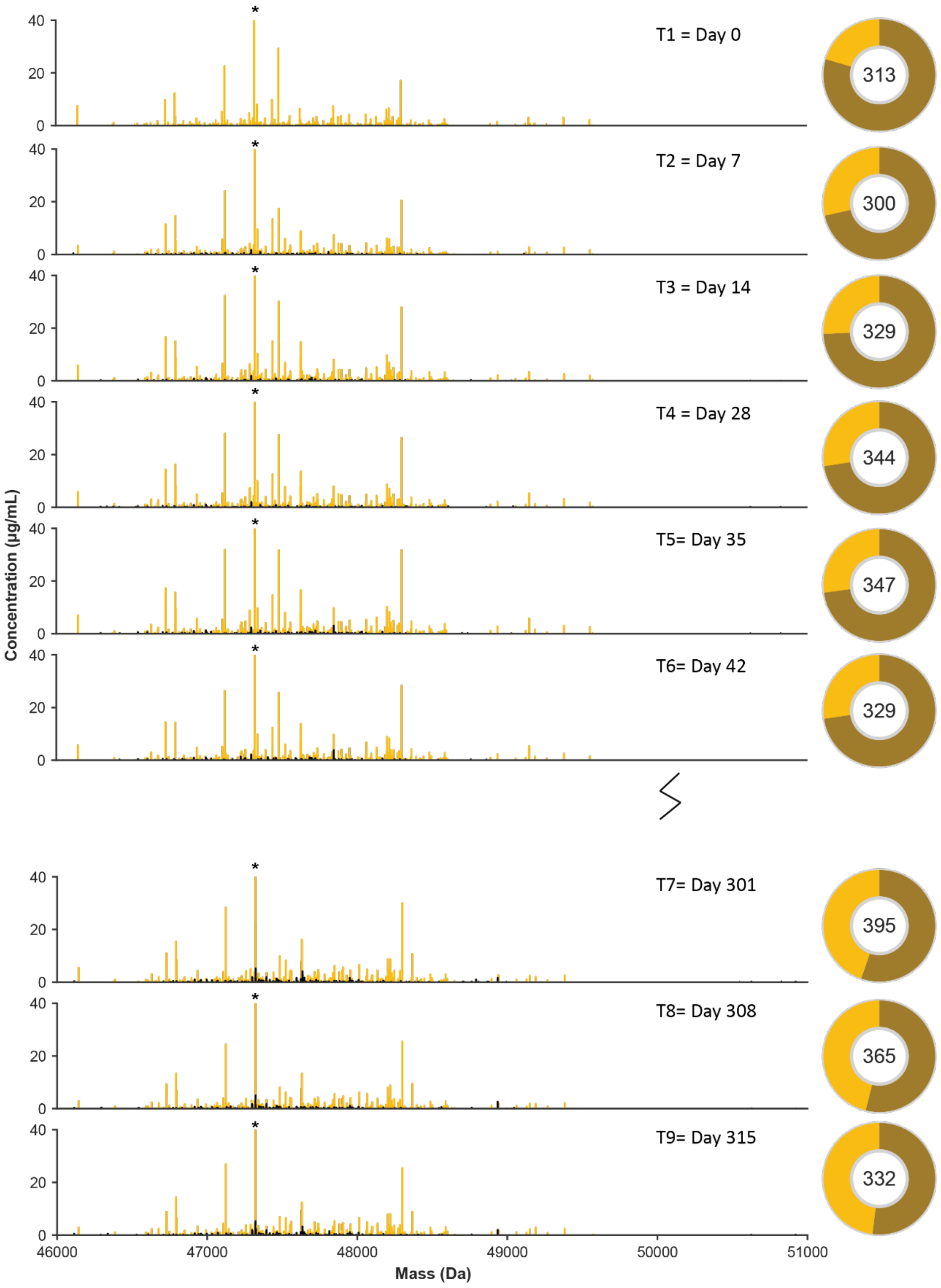

Fig. S1i-2. Longitudinal Fab clonal profiles of donor 19 at each of the 9 time points. Zoom into the profiles shown in SF1-1, y-axis multiplied by 25, to highlight specifically the lower concentration range. The peak annotated with an * is of the scale of the y-axis and is the high abundant clone visible in Figure 1i-1.

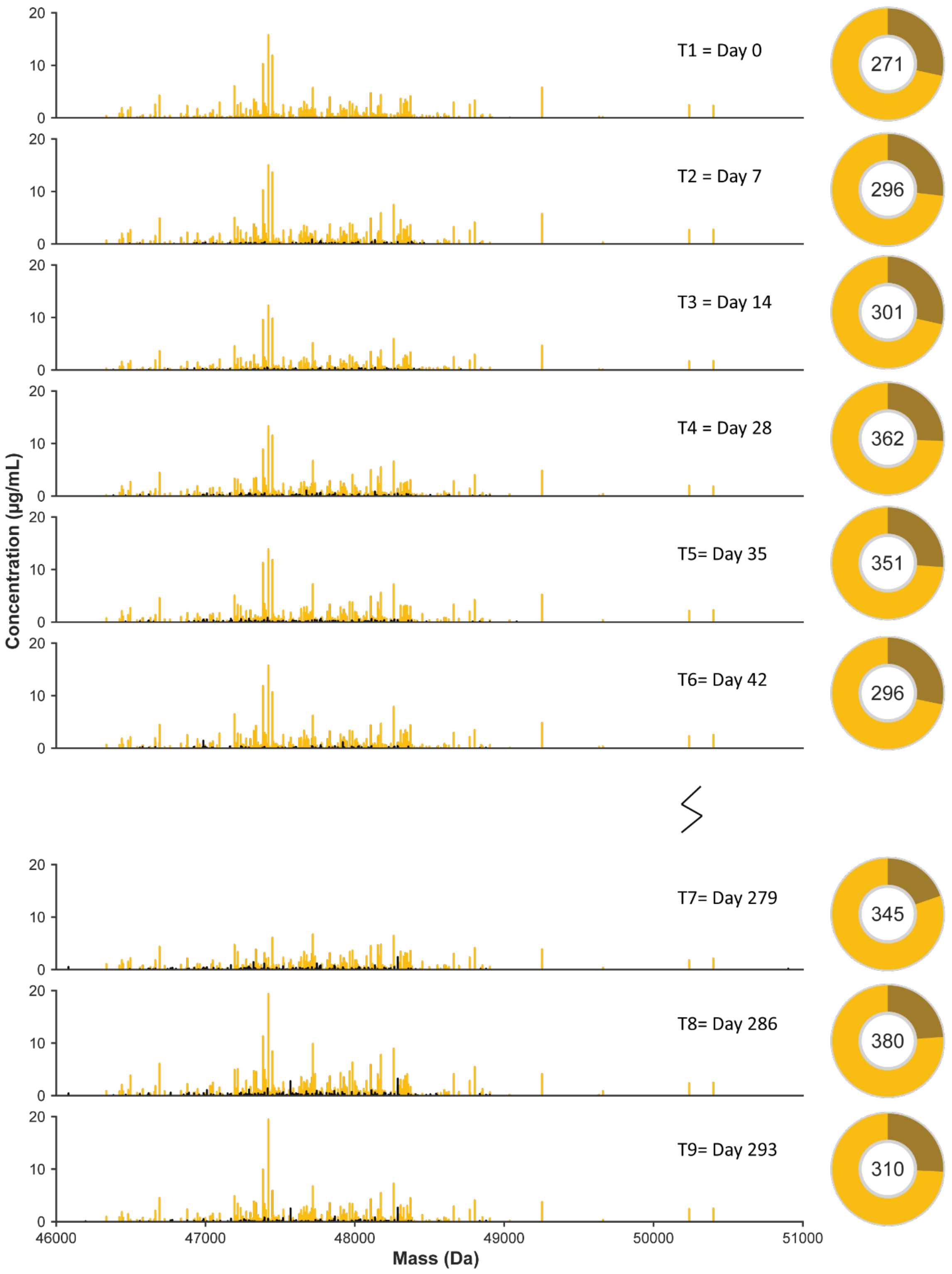

Fig. S1j. Longitudinal Fab clonal mass profiles of donor 22 at each of the 9 time points. Each peak represents a unique Fab at its detected mass and plasma concentration. The black clones are clones that were not present at Day 0. The pie charts display in the middle the total number of clones annotated, and relative contribution of the top 12 (Light brown) clones to the total IgG1 concentration (ocher yellow) in donor 22 at each time point.

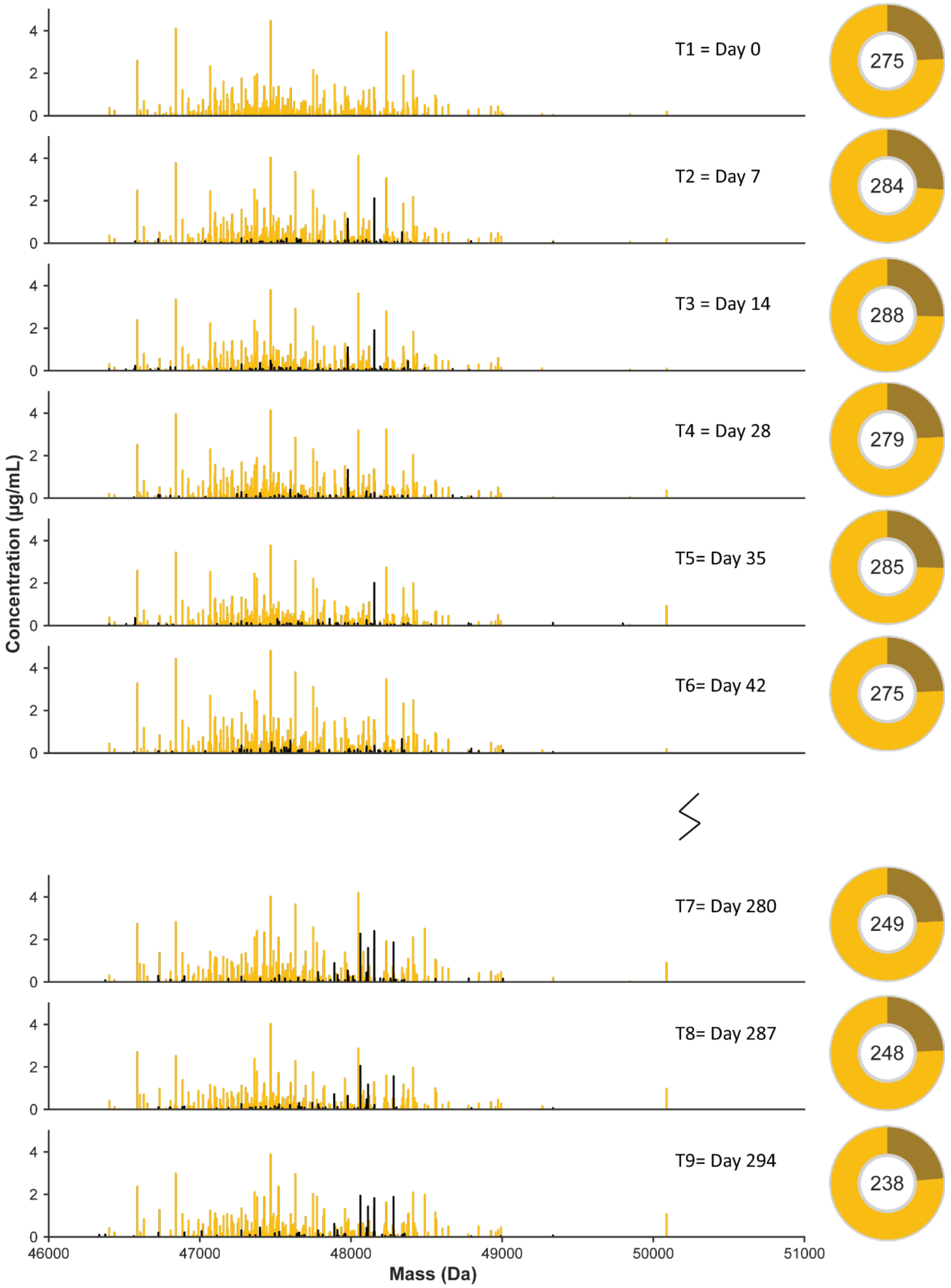

Fig. S1k. Longitudinal Fab clonal mass profiles of donor 26 at each of the 9 time points. Each peak represents a unique Fab at its detected mass and plasma concentration. The black clones are clones that were not present at Day 0. The pie charts display in the middle the total number of clones annotated, and relative contribution of the top 12 (Light brown) clones to the total IgG1 concentration (ocher yellow) in donor 26 at each time point.

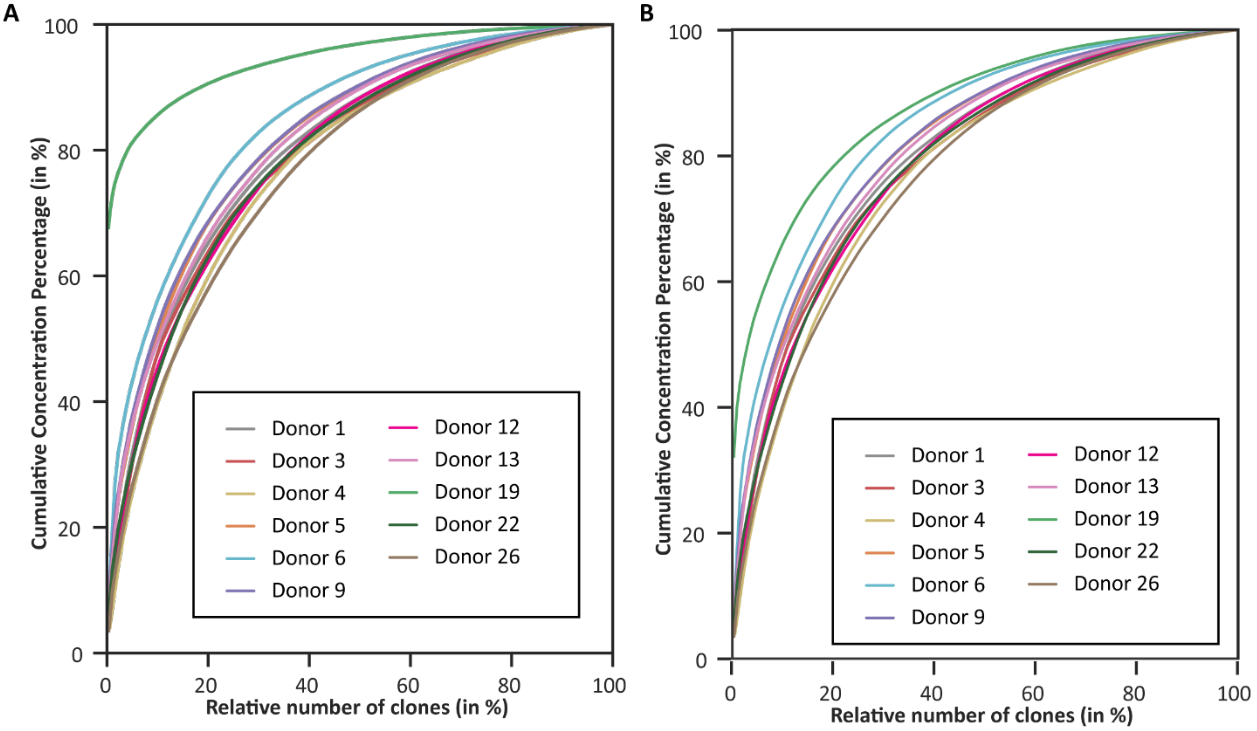

Fig. S2. Polydispersity of the IgG1 repertoires in healthy donors at T1 and T9. (A) Polydispersity of the IgG1 clonal repertoires of the different donors at T1. With on the X-axis the relative number of clones in %, and on the Y-axis the percentage of the total concentration that these % of clones are accounting for. All donors show a similar polydispersity, except for donor 19, where 1 clone accounts for ~70% of the total IgG1 concentration in that donor. **(B)** Polydispersity of the IgG1 clonal repertoires of the different donors at T9 (~1 year later compared to A). For all donors the polydispersity scores are highly comparable to those shown in A. Only donor 19 shows a substantial difference when comparing T1 and T9, which is accountable to the most abundant clone showing a relative decrease in concentration when comparing T0 and T9 (Supplementary Figure 1i-1 and 1i-2).

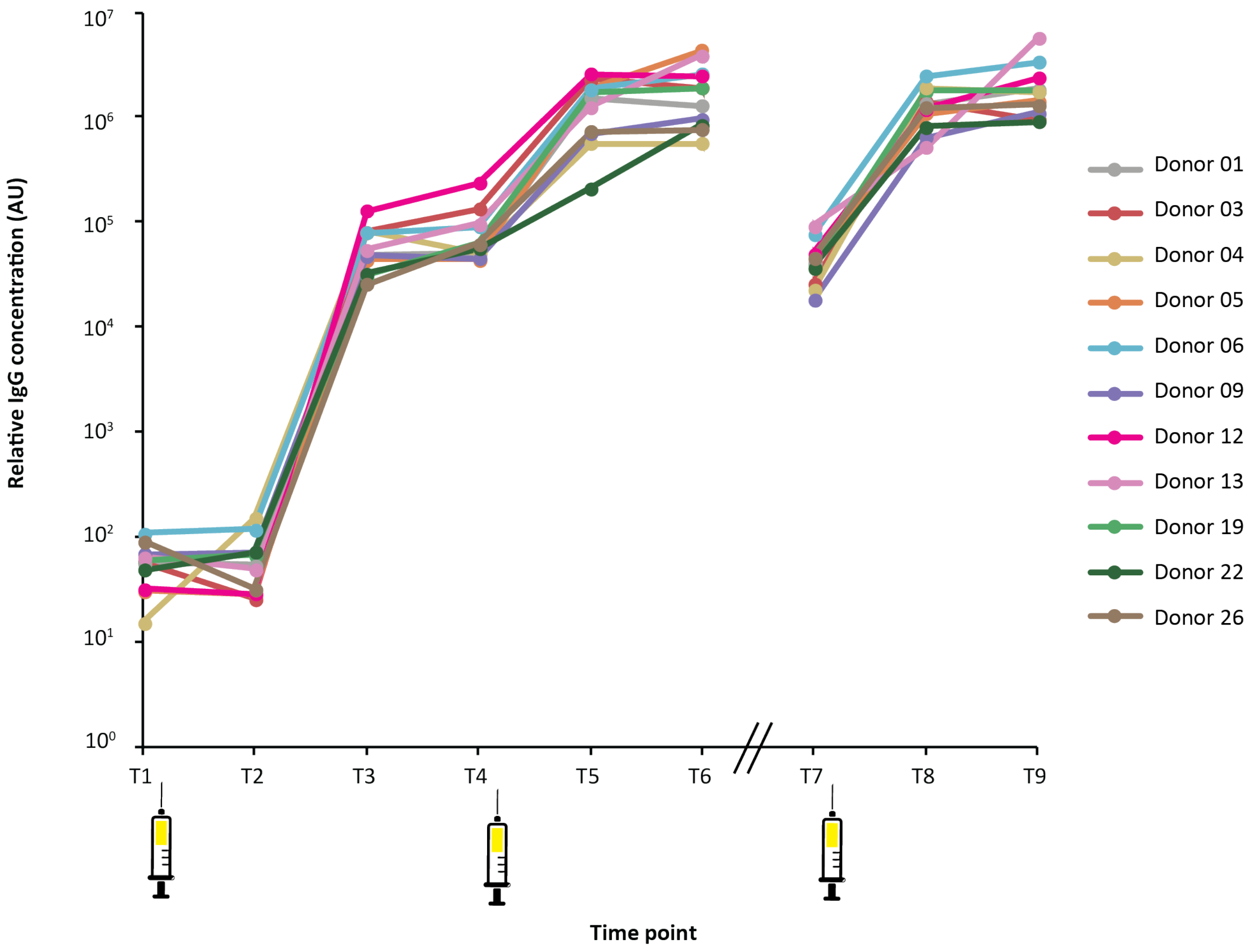

Fig S3. Total plasma IgG levels against the SARS-CoV-2 Spike S1. Anti- SARS-CoV-2 Spike S1 IgG levels were measured with a Luminex assay at the different time points after BNT162b2 vaccination. Plasma samples were serially diluted and the relative IgG concentration (AU) was determined by multiplying the half-maximal median fluorescence intensities with the corresponding dilution factor. Plasma samples were obtained at the day of vaccination as well as 7 and 14 days later. IgG response reflects the kinetics of a prime-boost immunization characterized by a slow and moderate primary response and a faster and stronger secondary response. Notably, this assays measure the cumulative response of all sub-classes of IgG.

Table S1. Detected total IgG1 concentration (in ug/mL) per sample using Fab clonal profiling.

		Time point								
		**1**	**2**	**3**	**4**	**5**	**6**	**7**	**8**	**9**
**Donor**	**1**	135	133	108	291	206	148	220	183	196
	**3**	46	131	97	108	75	72	106	122	117
	**4**	114	192	248	224	252	206	170	227	132
	**5**	248	190	239	223	201	309	197	204	215
	**6**	520	470	387	432	294	394	538	490	587
	**9**	522	622	483	544	542	615	617	606	728
	**12**	186	161	128	258	180	241	200	243	216
	**13**	189	[redacted]	155	149	205	193	311	260	277
	**19**	1141	849	1322	1236	1421	1156	862	708	680
	**22**	283	318	242	305	324	303	393	417	314
	**26**	127	132	122	131	122	157	141	114	121

Table S2. Comprehensive metadata for all study participants at all time points (Excel file).
